# Supplementary material for: Pervasive translation of circular RNAs driven by short IRES-like elements
Source: Nat Commun. 2022 Jun 29;13:3751. doi: 10.1038/s41467-022-31327-y (PMC9242994; doi:10.1038/s41467-022-31327-y)
Supplement: Supplementary file 1 — Supplementary Figures [file 41467_2022_31327_MOESM1_ESM.pdf]

# Summary of supplemental information

Supplementary Figure 1. Short elements can drive circRNA translation, related to Figure 1.

Supplementary Figure 2. circRNA can be translated from internal coding sequence, related to Figure 2.

Supplementary Figure 3. Functional analysis of identified *trans*-acting factors, related to Figure 3.

Supplementary Figure 4. Functional inference of putative circRNA-coded proteins, related to Figure 4.

Supplementary Figure 5. circRNA-coded proteins are hard to identify with low abundance that caused by quick degradation, related to Figure 4.

Supplementary Figure 6. Rolling circle translation of circRNAs in SH-SY5Y cells, related to Figure 5.

Supplementary Figure 7. Testing the IRES activity with a bicistronic reporter and circRNA reporters.

Supplementary Table 1. Primers and Probes used in this study, related to method part (see attached excel file)
Sheet1 Primers are used in this study.
Sheet2 Synthesized RNA probes for RNA affinity purification.
Sheet3 RNA probe sequences for northern blot

Supplementary Table 2. Enrichment score of each hexamer, related to Figure 1. (see attached excel file)

Supplementary Table 3. Identified *trans*-factors that bind to RNA probes, related to Figure 3. (see attached excel file)

Supplementary Table 4. Identified circORF-coded peptides by mass-spectrometry, related to Figure 4. (see attached excel file)

# Figures and Figure legends

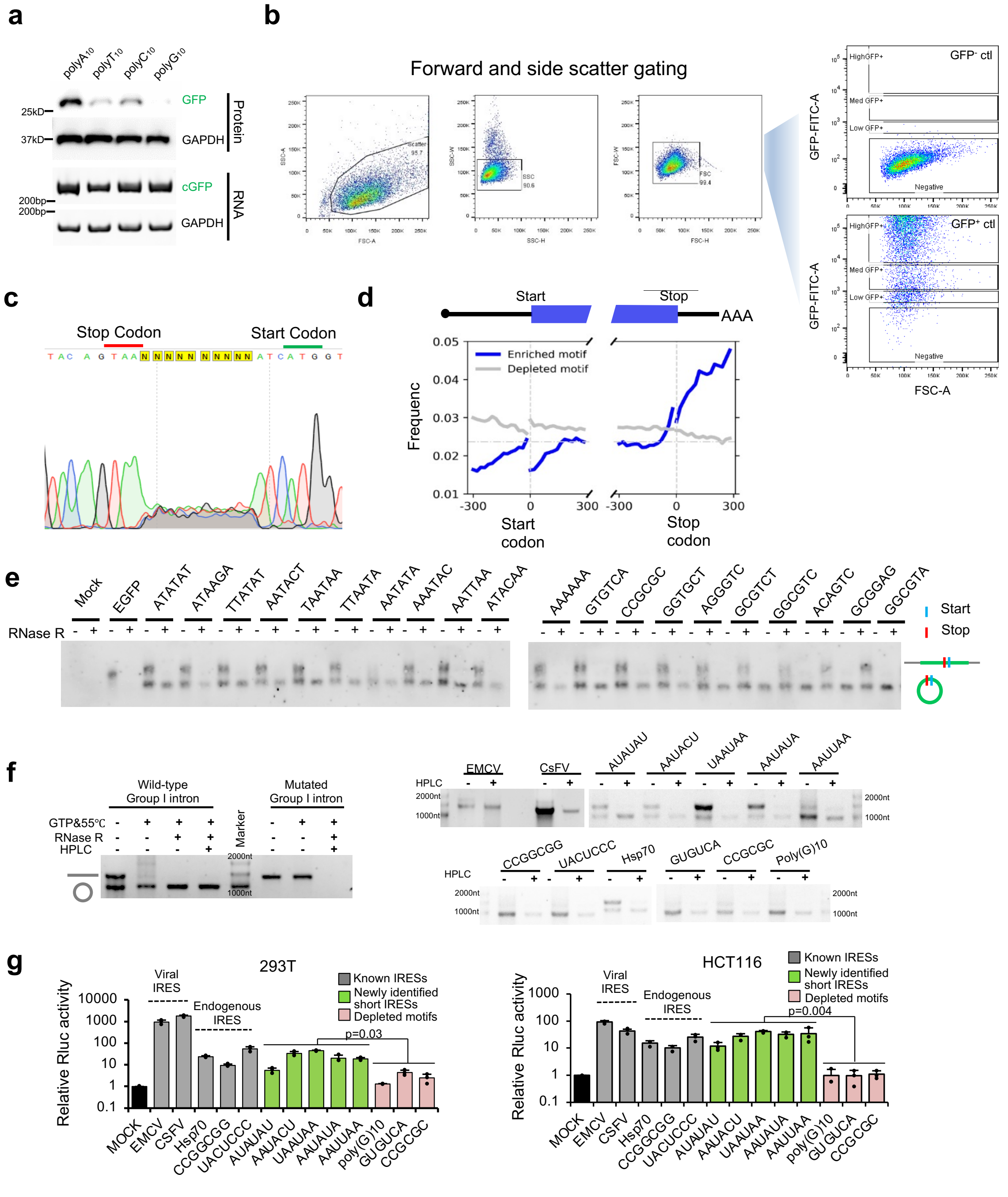


**Supplementary Figure 1. Short elements can drive circRNA translation.**

**(a)** CircRNA translation driven by polyN sequences. The pcircGFP plasmids inserted with different polyN$_{10}$ sequences were transfected into 293T cells, and samples were analyzed by western blot and RT-PCR 2 days after transfection. Source data are provided as a

Source Data file.

**(b)** Stepwise gating of single live cells in FACS. The green fluorescence of the cells after such gating is shown on Figure 1b. The wild-type 293T cells and the cells transfected with the pEGFP-C1 were used as negative and positive controls (GFP- ctl, GFP+ ctl) for the gating of green fluorescence during cell sorting (Figure 1b).

**(c)** Sequencing chromatogram around the inserted region in the random 10mer library.

**(d)** Position distribution of IRES-like hexamers in different regions of mRNAs.

**(e)** RNase R treatment and Northern blot analysis of circRNAs with enriched motifs (left) and depleted motifs (right). To cleavage the linear RNAs efficiently, RNA samples were heated to disrupt the secondary structures before RNase R treatment. Such samples were further analyzed by northern blot (please see details in method). Linear GFP indicates the entire GFP transcript from pEGFP-C1 vectors as a size marker.

**(f)** *In vitro* circRNA synthesis by self-splicing of group I intron. Linear RNAs were generated through *in vitro* transcription, and then circularized through self-splicing (details in method). The resulting RNAs were treated with RNase R and purified by HPLC (left panel). The HPLC purified circRNAs were verified by agarose gels (right panel).

**(g)** Translation of *in vitro* synthesized circRNAs. CircRNAs of luciferase containing known IRESs and newly identified IRES-like or depleted hexamers were generated through self-splicing of group I intron. These circRNAs were transfected into 293T and HCT116 cells. 24 hours after transfection, the cells were lysed for luminescence measurement using microplate reader. The P values of the differences between newly identified short IRESs and depleted motifs were calculated by two-sided Welch's t-test. Source data are provided as a Source Data file.

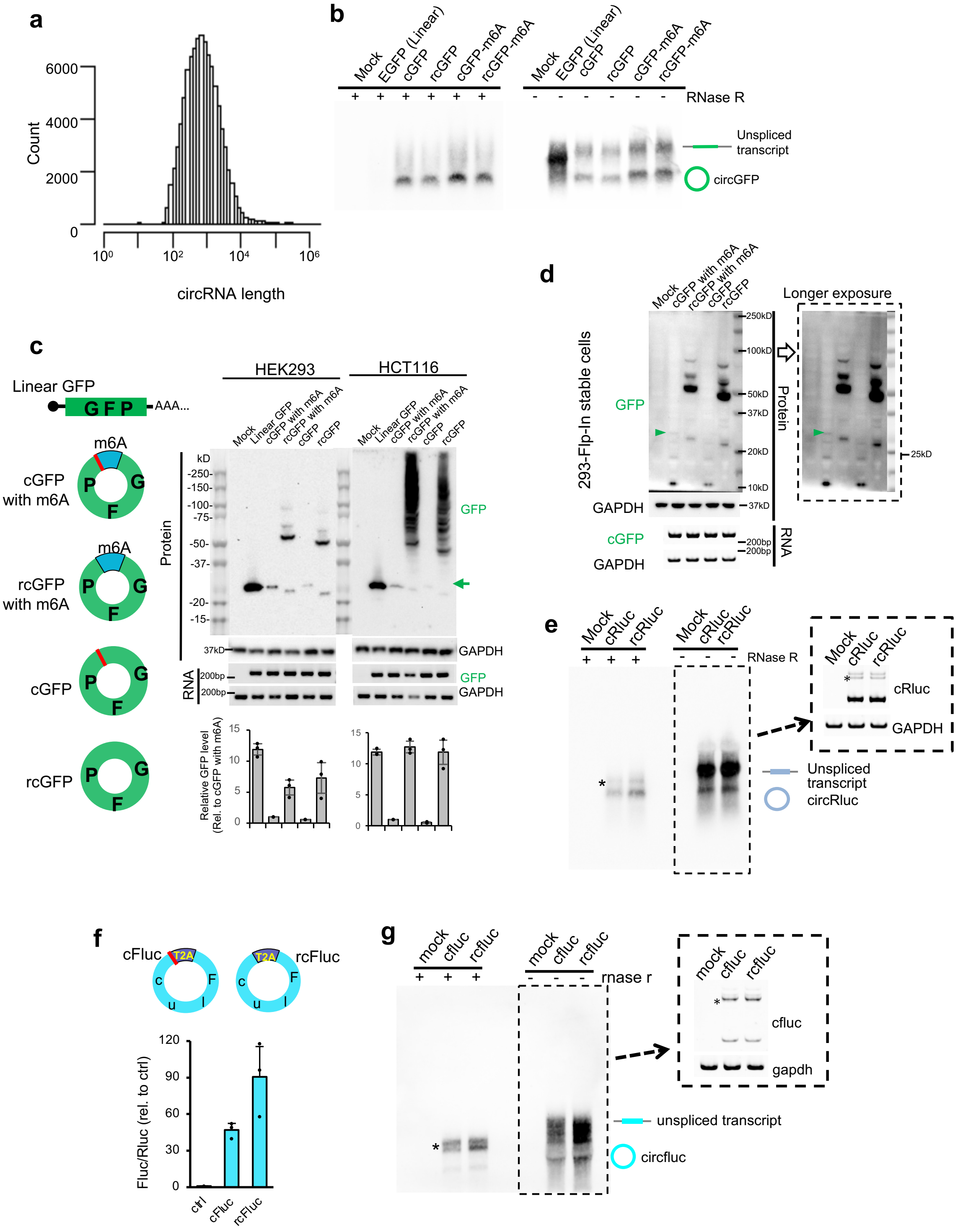

**Supplementary Figure 2. circRNA can be translated from internal coding sequence.**

Source data are provided as a Source Data file for panels **c, d and f.**

**(a)** Length distribution of all circRNAs from circBase.

**(b)** RNase R treatment and Northern blot analysis of the cGFP expression in Figure 2b.
**(c)** Testing translation of circRNAs in 293 cells and HCT116 cells. Similar to Figure 2b, the circRNA plasmids were transfected into additional types of cells, and were analyzed by western blot at 2 days after transfection (right panel). The green arrow indicates the full-length GFP, and the smears at the top of gels indicate rolling circle translation products. The bar graph represents the relative GFP levels normalized by the RNA level.
**(d)** Western blot analysis of circRNA translation of 293T Flp-In stable cells. DNA fragments containing split GFP exon were inserted into pcDNA5/FRT vectors, and then were co-transfected with pOG44 into 293T-Flp-In cells at 1:9 ratio followed by hygromycin selection. The stable cells were collected to test circRNA expression and translation. The green arrows indicate the full-length GFP protein.
**(e)** RNase R treatment and Northern blot analysis of cRluc expression in Figure 2c. Dashed boxes: circRluc expression is detected by RT-PCR using divergent primers. The asterisks indicate a minor back splice isoform that used an alternative 3' ss.
**(f)** circRNA translation without inserted IRES-like elements. Top: schematic diagram of two Fluc circRNAs, where the Fluc ORF was split into two parts in reverse order and thus can form an intact ORF after back-splicing. A T2A peptide was inserted into the circRNAs so that the product of rcORF can be cleaved into smaller fragments. Bottom: dual luciferase assay of cFluc and rcFluc. Control and circular Fluc plasmids were co-transfected with Rluc reference reporter into 293T cells. At 48 hours after transfection, cells were lysed, and luminescence was measured by microplate reader.
**(g)** RNase R treatment and Northern blot analysis of cFluc expression in Supplementary Figure 2e. Dashed boxes: circFluc expression is detected by RT-PCR using divergent primers. The asterisks indicate a minor back splice isoform that used an alternative 3' ss.

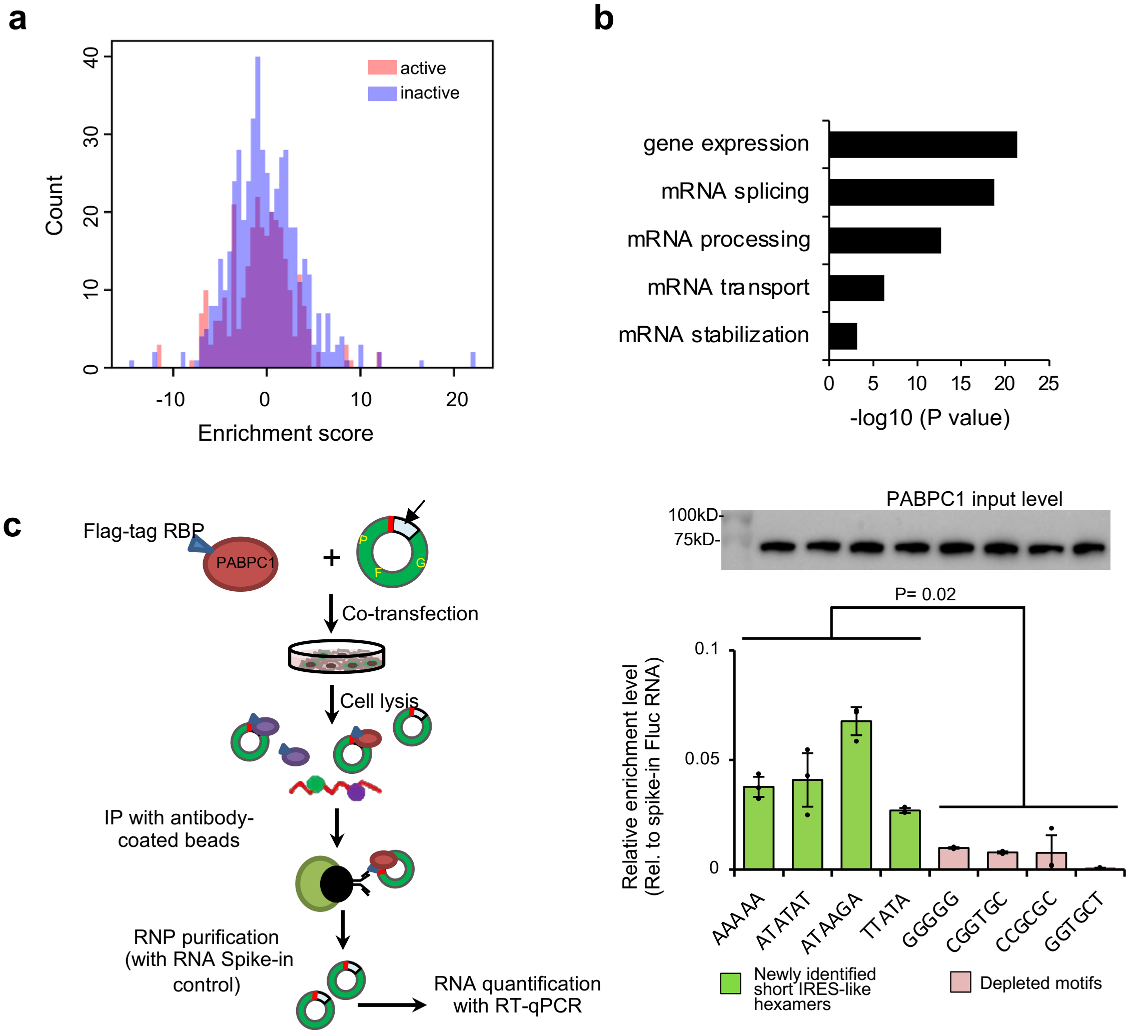


**Supplementary Figure 3. (a)** The 18S rRNA active hexamers (red bar) or inactive hexamers (blue bar) have no distribution biases in the IRES-like elements. The enrichment scores were calculated according to the sequences found in green cells *vs.* dark cells in our screen of IRES-like sequences.

**(b)** Functional analyses of the identified *trans*-acting factors that bind the IRES-like elements.

**(c)** The association of PABPC1 with IRES-like hexamers and depleted motifs was tested by RIP analysis using Anti-FLAG M2 antibody (Mouse). After IP, the presence of

PABPC1 in the beads was confirmed by Western blot analysis using DYKDDDDK antibodies (Rabbit). The relative enrichment of RNAs was assessed by RT-qPCR, and normalized to the levels of spike in Fluc RNA in each sample. Source data are provided as a Source Data file.

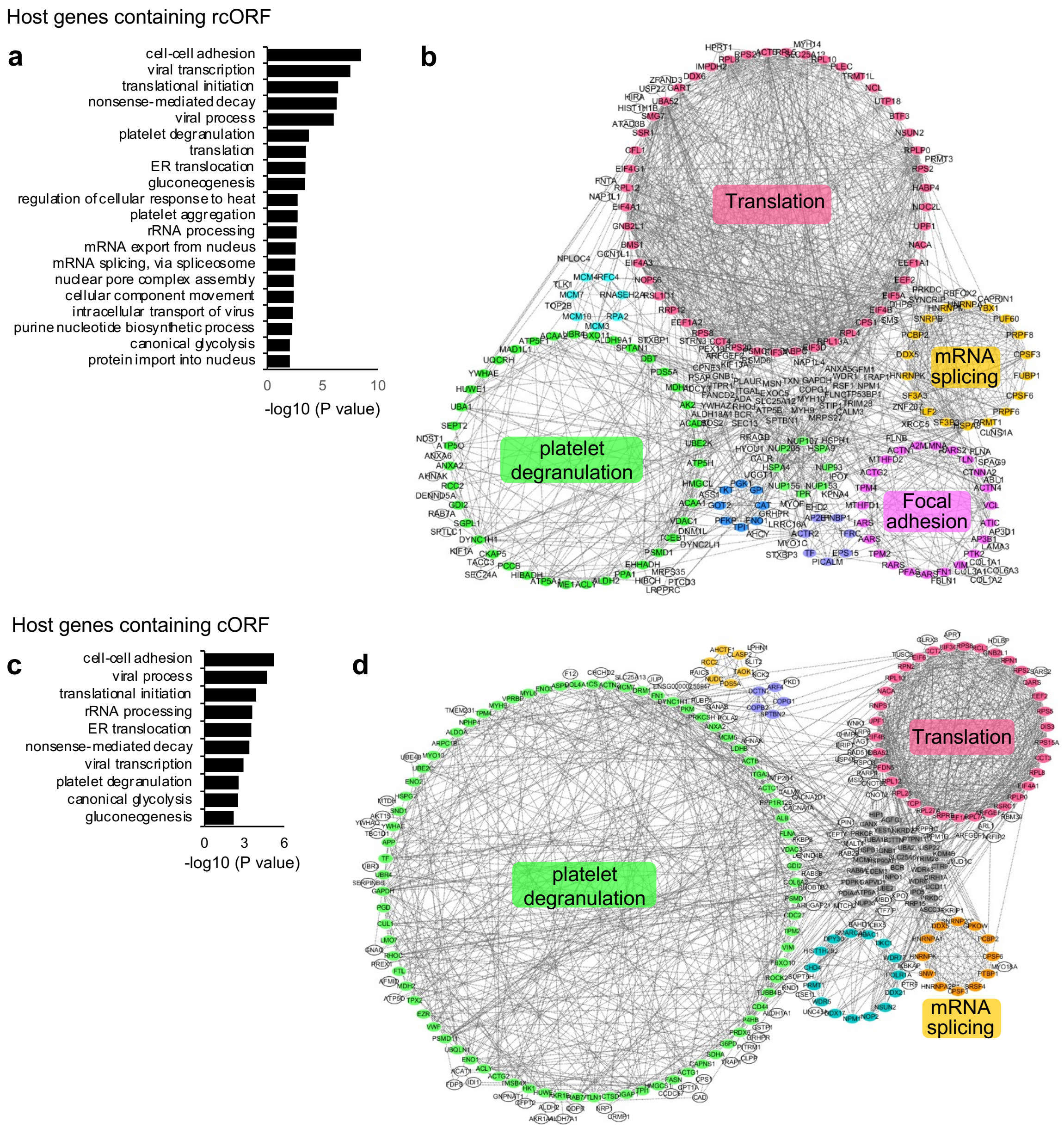


**Supplementary Figure 4. Functional inference of putative circRNA-coded proteins.**

**(a) and (c).** Enrichment of biological processes of host genes containing translatable circRNAs with rcORFs (**a**) or cORFs (**c**).

**(b)** and **(d)** Protein-protein association networks of host genes containing translatable circRNAs with rcORFs (**b**) or cORFs (**d**). The STRING database was searched by host genes containing translatable circRNAs using the default parameters (with high confidence, considering of all interaction evidences and discarding disconnected nodes), and the resulting networks were clustered using MCODE tool.

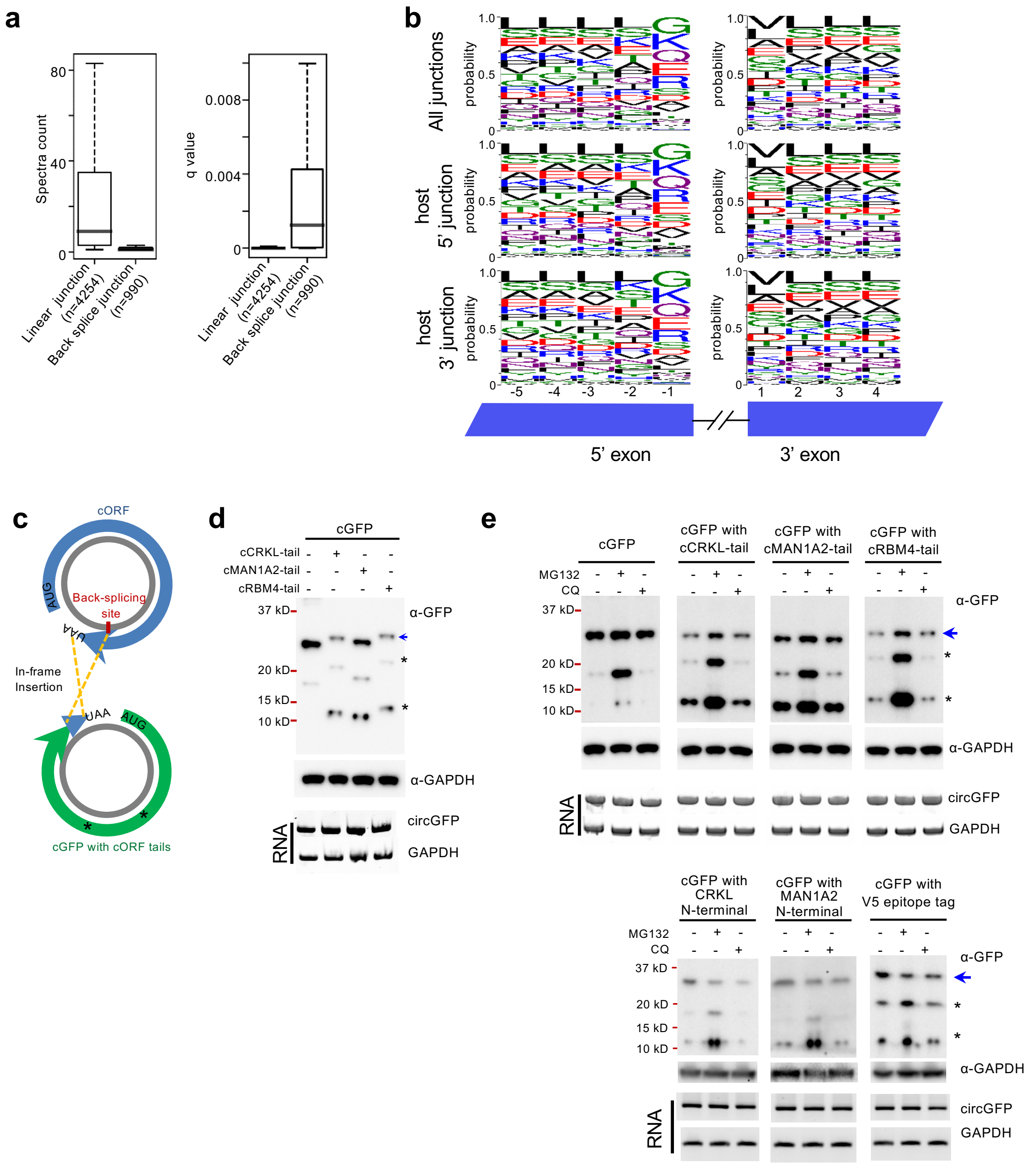

**Supplementary Figure 5. circRNA-coded proteins are hard to identify with low abundance that caused by quick degradation.**

**(a)** The spectra number and q-value of peptides across back splice junctions and corresponding adjacent linear junctions. Both box plots have the median as the center and the first and third quartiles as the upper and lower edges of the box. The upper line is the third quartile plus 1.5 × interquartile range, and the lower line is the first quartile minus 1.5×interquartile range.

**(b)** Amino acid composition of peptides across splice junctions. All junctions indicate the junction regions of all the mRNAs, host 5' junctions or 3' junctions indicate the 5' junctions or 3' junctions at the host gene of translatable circRNA.

**(c)** Schematic diagram of the circGFP with endogenous circRNA-coded tails. The circRNA contains a predicted reading frame that includes the overlapped N-terminal region (i.e. it is overlapped with the reading frame of the host gene) and the shifted C-terminal region (i.e. its reading frame is shifted after the back-splicing site, named as circRNA-coded tail). This frame-shifted coding region is inserted at the C-terminal of the GFP within the circGFP.

**(d)** The circGFPs with circRNA-coded tails are unstable. The circGFPs with different circRNA-coded tails (circCRKL-tail, circMAN1A2-tail, or cRBM4-tail) were transfected into 293T cells. Samples were analyzed by western blot and RT-PCR at 48 hours after transfection. The blue arrow indicates the full length of GFP protein with or without inserted tails, the asterisks indicate the truncated GFP proteins that are translated from the internal start codons within the GFP coding region. Source data are provided as a Source Data file.

**(e)** CircRNA-coding tails induce rapid protein degradation through proteasome pathway. The circGFPs with different circRNA-coded tails (circCRKL-tail, circMAN1A2-tail, or cRBM4-tail) and N-terminal peptide of the same circRNA-coded protein (CRKL N-terminal or MAN1A2 N-terminal) or V5-epitope tag were transfected into 293T cells. Then the transfected cells were treated with 10 μM MG132 for 2 hours, or 10 μM chloroquine for 4 hours before cell collection. Source data are provided as a Source Data file.

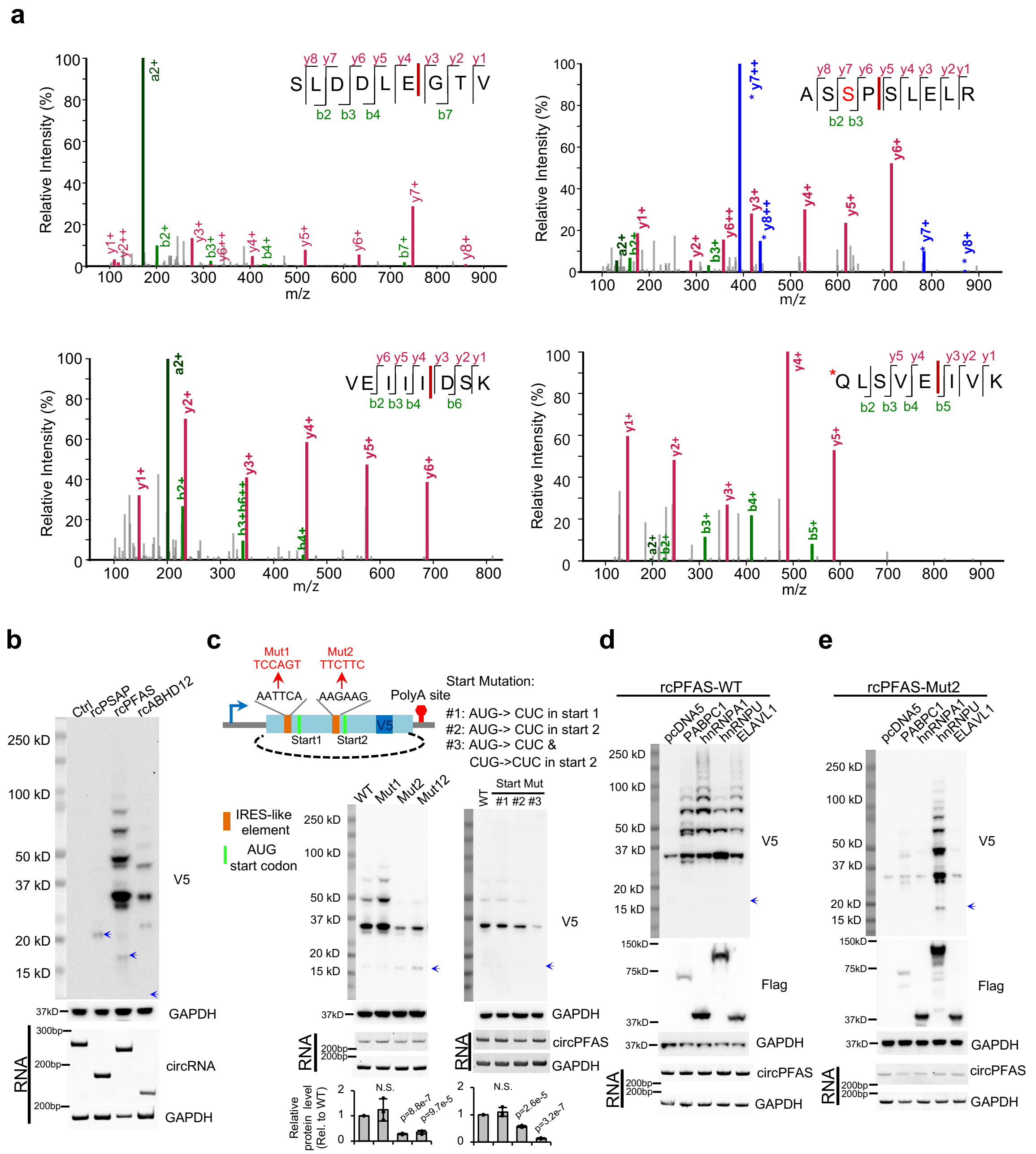


**Supplementary Figure 6. Rolling circle translation of circRNAs in SH-SY5Y cells.**

**(a)** Additional examples of the MS spectra for circRNA-encoded peptides across the back splice junction of the human circRGS12 (SLDDLEGTV), circPITRM1 (ASSPSLELR), circMALT1 (VEIIIDSK), and circHUWE1 (QLSVEIVK). The annotated b- and y-ions are marked in red and green color, respectively.

**(b)** The RNA and protein expression of rcORF translation reporters in SH-SY5Y cells. The cells were collected and analyzed using same procedures as described in Figure 5c.

Source data are provided as a Source Data file.

**(c)** The translation of rcPFAS is reduced by mutations in IRES-like elements or the start codons of the circPFAS in SH-SY5Y cells. The RNAs and proteins were detected using similar procedure as described in Figure 5e. The bar graph represents the quantification of protein levels relative to GAPDH. The protein levels were also normalized to the RNA (N.S.: not significant). Source data are provided as a Source Data file.

**(d)** *Trans*-factors promote rolling circle translation of endogenous rcORFs in SH-SY5Y cells. The RNAs and proteins were detected using similar procedure as described in Figure 5f. Source data are provided as a Source Data file.

**(e)** The circRNA with mutated IRES-like hexamer AAGAAG (Mut2) was co-expressed with same set of *trans*-acting factors, and the production of rolling circle translation in SH-SY5Y cells were measured using same experimental conditions described in Figure 5g. Source data are provided as a Source Data file.

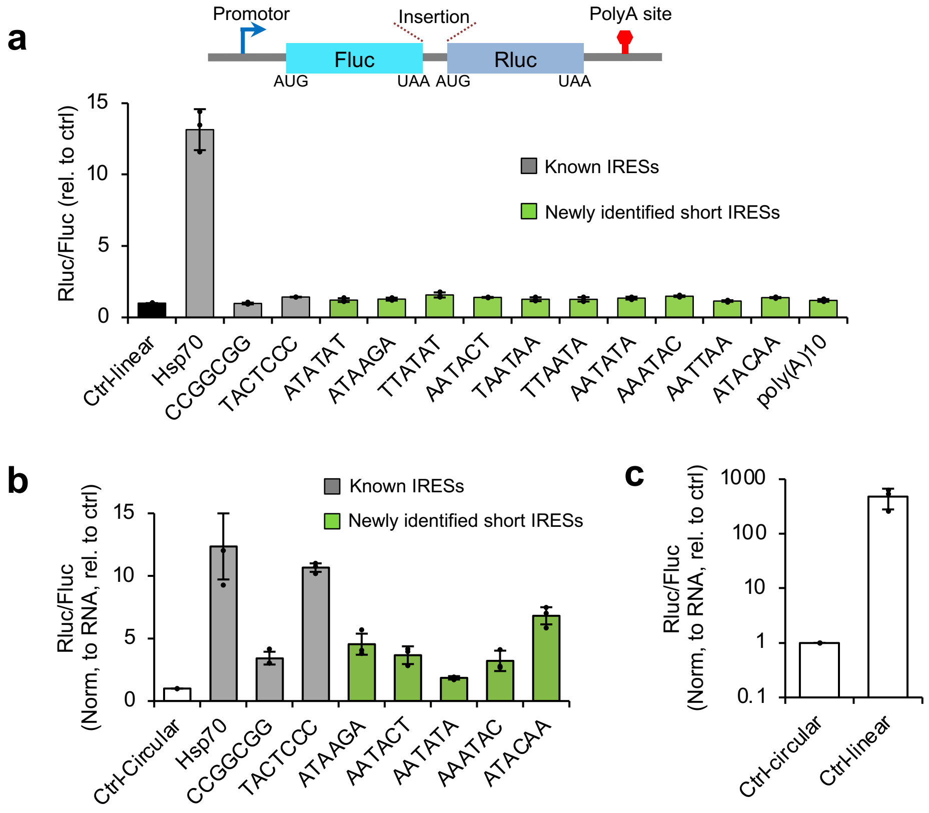


**Supplementary Figure 7. Testing the IRES activity with a bicistronic reporter and circRNA reporters.**

**(a)** Standard bicistronic reporters containing the known endogenous IRESs and newly identified IRES-like elements were transfected into 293T cells. At 48 hours after transfection, cells were lysed, and luminescence was measured by microplate reader to calculate the relative activity of two luciferases translated from different ORFs. The empty control reporter contains only the short cloning site (GAGACGACACCGTCTCATC). Source data are provided as a Source Data file.

**(b)** The IRES activity of short elements tested in circRluc. The circRNA reporters encoding Rellina luciferase with known endogenous IRESs and IRES-like elements were transfected into 293T cells. The RNA levels were detected by qPCR, and the luminescence was measured using same experimental conditions described in Supplementary Figure 7a. The empty control reporter contains the same short cloning site. Source data are provided as a Source Data file.

**(c)** Comparison of translation background between linear bicistronic and circular

reporters. The empty controls of both reporters were transfected into 293T cells, and the RNA levels and luminescence were measured using same experimental conditions described in Supplementary Figure 7b. Source data are provided as a Source Data file.
