# Supplementary material for: Pervasive translation of circular RNAs driven by short IRES-like elements
Source: Nat Commun. 2022 Jun 29;13:3751. doi: 10.1038/s41467-022-31327-y (PMC9242994; doi:10.1038/s41467-022-31327-y)
Supplement: Supplementary file 3 — Description of Additional Supplementary Files [file 41467_2022_31327_MOESM3_ESM.pdf]

Description of Additional Supplementary Files

File Name: Supplementary Data 1
Description: Primers and Probes used in this study, related to method part
Sheet1 Primers are used in this study.
Sheet2 Synthesized RNA probes for RNA affinity purification.
Sheet3 RNA probe sequences for northern blot

File Name: Supplementary Data 2
Description: Enrichment score of each hexamer, related to Figure 1.

File Name: Supplementary Data 3
Description: Identified *trans*-factors that bind to RNA probes, related to Figure 3.

File Name: Supplementary Data 4
Description: Identified circORF-coded peptides by mass-spectrometry, related to Figure 4.
