# Supplementary material for: Pervasive translation of circular RNAs driven by short IRES-like elements
Source: Nat Commun. 2022 Jun 29;13:3751. doi: 10.1038/s41467-022-31327-y (PMC9242994; doi:10.1038/s41467-022-31327-y)
Supplement: Supplementary file 8 — Reporting Summary [file 41467_2022_31327_MOESM8_ESM.pdf]

nature portfolio

Corresponding author(s): Zefeng Wang

Last updated by author(s): May 29, 2022

Reporting Summary

Nature Portfolio wishes to improve the reproducibility of the work that we publish. This form provides structure for consistency and transparency in reporting. For further information on Nature Portfolio policies, see our Editorial Policies and the Editorial Policy Checklist.

Statistics

For all statistical analyses, confirm that the following items are present in the figure legend, table legend, main text, or Methods section.

n/a	Confirmed	
☐	☒	The exact sample size (*n*) for each experimental group/condition, given as a discrete number and unit of measurement
☐	☒	A statement on whether measurements were taken from distinct samples or whether the same sample was measured repeatedly
☐	☒	The statistical test(s) used AND whether they are one- or two-sided<br>*Only common tests should be described solely by name; describe more complex techniques in the Methods section.*
☒	☐	A description of all covariates tested
☒	☐	A description of any assumptions or corrections, such as tests of normality and adjustment for multiple comparisons
☐	☒	A full description of the statistical parameters including central tendency (e.g. means) or other basic estimates (e.g. regression coefficient) AND variation (e.g. standard deviation) or associated estimates of uncertainty (e.g. confidence intervals)
☐	☒	For null hypothesis testing, the test statistic (e.g. *F*, *t*, *r*) with confidence intervals, effect sizes, degrees of freedom and *P* value noted<br>*Give P values as exact values whenever suitable.*
☒	☐	For Bayesian analysis, information on the choice of priors and Markov chain Monte Carlo settings
☒	☐	For hierarchical and complex designs, identification of the appropriate level for tests and full reporting of outcomes
☒	☐	Estimates of effect sizes (e.g. Cohen's *d*, Pearson's *r*), indicating how they were calculated

Our web collection on statistics for biologists contains articles on many of the points above.

Software and code

Policy information about availability of computer code

Data collection

The following system was used for data collection:
1. Flow cytometry: BD FACSAria II.
2. Gel and blot imager: BioRad ChemiDoc Touch.
3. Luminescence Detection: Bio-Tek synergy H1.
4. RNA-seq: Illumina Hiseq 2500.

Data analysis

The following softwares were used for data analysis:
1. Mascot: for protein identification from Mass-spectrometry data.
2. Image Lab : for capturing and exporting the images of gels and blots.
3. FlowJo v10.5.0 : for flow cytometry data.
4. Adobe Photoshop CC 2017 : for making images.
5. Powerpoint 2016 : for making figures.
6. Excel 2016: for recording readings and final datas.
7. python 3.7.2: data analysis, image plot
8. CLUSTALW2: motif clustering
9. Weblogo 3: motif logo generating
10. STRING 8: protein–protein interaction networks analysis
11. MCOD(v1.6.1)E: network clustering
12. pFind (v3.1.3): Mass spectrometry data analysis
13. blastp (v2.6.0+): detect the homologues of circRNA-coded ORFs
14. CIRI2 v2.0.6: full length circRNA analysis

For manuscripts utilizing custom algorithms or software that are central to the research but not yet described in published literature, software must be made available to editors and reviewers. We strongly encourage code deposition in a community repository (e.g. GitHub). See the Nature Portfolio guidelines for submitting code & software for further information.

Data

Policy information about availability of data

All manuscripts must include a data availability statement. This statement should provide the following information, where applicable:

- Accession codes, unique identifiers, or web links for publicly available datasets
- A description of any restrictions on data availability
- For clinical datasets or third party data, please ensure that the statement adheres to our policy

Four published circRNA datasets (Figure 2a, and 4a) were retrieved from circBase (http://circbase.org/). The full-length circRNA dataset (Figure 4b and 4d) was obtained from the published ribominus RNA-seq data that was generated from the RNase R treated RNAs of HeLa cells (BioProject database of Genbank, accession number PRJNA266072).
Two human comprehensive proteome datasets (Figure 4d-g, and Supplementary Figure 5a-b) were obtained from Bekker-Jensen, DB, et al, and Kim, MS, et al. & Pinto, SM, et al., which in turn rely on freely available data obtained from PRIDE Archive (accession number: PXD004452, and PXD000561)
The RNA-seq data generated in this study have been deposited in the GEO database under accession code GSE152560.
Source data are provided with this paper.

Human research participants

Policy information about studies involving human research participants and Sex and Gender in Research.

Reporting on sex and gender	n/a
Population characteristics	n/a
Recruitment	n/a
Ethics oversight	n/a

Note that full information on the approval of the study protocol must also be provided in the manuscript.

Field-specific reporting

Please select the one below that is the best fit for your research. If you are not sure, read the appropriate sections before making your selection.

☒ Life sciences ☐ Behavioural & social sciences ☐ Ecological, evolutionary & environmental sciences

For a reference copy of the document with all sections, see nature.com/documents/nr-reporting-summary-flat.pdf

Life sciences study design

All studies must disclose on these points even when the disclosure is negative.

Sample size

For all decamers, there are four possible bases in each of the ten positions, and thus there would be 4^{10} (=1,048,576, ~1 million) all hexamers in the entire sequence space. To cover all possible decamers, 2 million E. coli clones (~2-fold coverage) were collected and used for extracting the decamer plasmid library. During sorting, in total 122 million cells were sorted, we collected 4 million cells without GFP fluorescence (negative controls), 13 million cells with low GFP fluorescence, 5 million cells with medium GFP fluorescence and 0.5 million cells with high GFP.

Data exclusions	No data were excluded.
Replication	For all the experiments, three separate biological replicates were performed for each of the cell lines.
Randomization	No randomization was performed for the current study as there are not multiple experimental groups in this study. The experiments were performed under same conditions, and the results are objective and not rely on subjective judgment.
Blinding	Blinding was not required for this study because there were no data exclusions and conclusions were made on objective quantitative analysis of data.

Reporting for specific materials, systems and methods

We require information from authors about some types of materials, experimental systems and methods used in many studies. Here, indicate whether each material, system or method listed is relevant to your study. If you are not sure if a list item applies to your research, read the appropriate section before selecting a response.

Materials & experimental systems

n/a	Involved in the study
☐	☒ Antibodies
☐	☒ Eukaryotic cell lines
☒	☐ Palaeontology and archaeology
☒	☐ Animals and other organisms
☒	☐ Clinical data
☒	☐ Dual use research of concern

Methods

n/a	Involved in the study
☒	☐ ChIP-seq
☐	☒ Flow cytometry
☒	☐ MRI-based neuroimaging

Antibodies

Antibodies used

Primary antibody used are:
Living Colors® A.v. Monoclonal Antibody (JL-8) (Takara Bio Clontech, 632381,mouse), V5-Tag (D3H8Q) Rabbit mAb (Cell signaling technology: 13202S), Monoclonal ANTI-FLAG® M2 antibody produced in mouse (Sigma: F1804-1MG), DYKDDDDK Tag (D6W5B) Rabbit mAb (Cell signaling technology: 14793S), HRP-conjugated GAPDH Monoclonal antibody (Proteintech: HRP-60004).
Secondary antibodies used are:
Anti-mouse IgG, HRP-linked Antibody (Cell signaling technology: 7076S) Anti-rabbit IgG, HRP-linked Antibody (Cell signaling technology: 7074S)

Validation

All the antibodies were validated by manufacturers.
Living Colors® A.v. Monoclonal Antibody (JL-8) (Takara Bio Clontech, 632381,mouse): The Living Colors ZsGreen Monoclonal Antibody was raised in mouse against recombinant full-length Zoanthus sp. green fluorescent protein (ZsGreen), and Protein A-purified.
V5-Tag (D3H8Q) Rabbit mAb (Cell signaling technology: 13202S): V5-Tag (D3H8Q) Rabbit mAb was raised in rabbit ,and recognizes transfected levels of recombinant protein containing the V5 epitope tag.
Monoclonal ANTI-FLAG® M2 antibody produced in mouse (Sigma: F1804-1MG): The ANTI-FLAG M2 mouse, affinity purified monoclonal antibody binds to fusion proteins containing a FLAG peptide sequence. The antibody recognizes the FLAG peptide sequence at the N-terminus, Met-N-terminus, C-terminus, and internal sites of the fusion protein.
DYKDDDDK Tag (D6W5B) Rabbit mAb (Cell signaling technology: 14793S): DYKDDDDK Tag (D6W5B) Rabbit mAb detects exogenously expressed DYKDDDDK proteins in cells. The antibody recognizes the DYKDDDDK peptide, which is the same epitope recognized by Sigma's Anti-FLAG® antibodies, fused to either the amino-terminus or carboxy-terminus of the target protein.
HRP-conjugated GAPDH Monoclonal antibody (Proteintech: HRP-60004): HRP-60004 targets GAPDH in WB, IHC, IF applications and shows reactivity with human, mouse, rat, zebrafish, plant samples.

Eukaryotic cell lines

Policy information about cell lines and Sex and Gender in Research

Cell line source(s)	HEK293, HEK293T, Hela, HCT116 and SH-SY5Y cells were kindly provided by Cell Bank, Chinese Academy of Sciences.
Authentication	Cell lines were not authenticated.
Mycoplasma contamination	Cell lines were not tested for mycoplasma.
Commonly misidentified lines (See ICLAC register)	None used.

Flow Cytometry

Plots

Confirm that:

☒ The axis labels state the marker and fluorochrome used (e.g. CD4-FITC).

☒ The axis scales are clearly visible. Include numbers along axes only for bottom left plot of group (a 'group' is an analysis of identical markers).

☒ All plots are contour plots with outliers or pseudocolor plots.

☒ A numerical value for number of cells or percentage (with statistics) is provided.

Methodology

Sample preparation	The random decamer plasmid library was transfected into 293T cells (20 µg/ per 15 cm dish) by using lipofectamine 2000 (Invitrogen). To cover the entire decamer space, totally 10x 15 cm dishes were used. Cells were collected for FACS sorting at 48 hours after transfection.
Instrument	BD FACSAria II
Software	Cytometry data was collected in FACSDiva (BD) and analysed in FlowJo.
Cell population abundance	74.2% cells without GFP fluorescence (negative controls), 9.1% cells with low GFP fluorescence, 4.7% cells with medium GFP fluorescence and 0.4% cells with high GFP.
Gating strategy	FSC-A vs SSC-A was used to select 293T cells (excluding very small and very large particles). Two round selections of singlets were used by SSC-W vs FSC-H and FSC-W vs FSC-H. FSC-A vs FITC-A were used to select GFP-positive cells.

☒ Tick this box to confirm that a figure exemplifying the gating strategy is provided in the Supplementary Information.
